# Perioperative Management of Patients With Ankylosing Spondylitis Undergoing Spine Surgery

**DOI:** 10.3389/fphar.2020.01017

**Published:** 2020-07-09

**Authors:** Arman Zakaryan, Knarik Ginosyan

**Affiliations:** ^1^ Department of Neurosurgery, Yerevan State Medical University after Mkhitar Heratsi, Yerevan, Armenia; ^2^ Department of Rheumatology, Yerevan State Medical University after Mkhitar Heratsi, Yerevan, Armenia

**Keywords:** ankylosing spondylitis, spine surgery, spine deformity, perioperative management

Ankylosing spondylitis (AS) is the most frequent type of seronegative spondyloarthropathy, which mainly involves the axial spine. In advanced cases, the chronic inflammatory process can cause fibrosis and calcification, which leads to loss of flexibility and fusion of the vertebrae, resembling a “bamboo”, with a fixed posture, generally known as chin-on-chest deformation.

The functional limitations of this particular deformity are significant: the patients are unable to look forward, make visual contact, and have difficulty eating. All these factors with typically concomitant osteoporosis will significantly increase the risk of spinal injury with severe instability, deformity, and, most importantly, deteriorating neurological function or paralysis requiring spinal surgery. Common indications for spinal intervention on patients with AS are:

- Uncontrolled, severe and continuous back and neck pain
- Deteriorating neurological deficit caused by spinal deformity
- Unstable spinal fracture
- Inability to hold the head up and see horizontally
- Serious difficulties in eating and drinking because of spinal deformity.

Spinal surgery on these patients is related to a greater risk of surgical and anesthesia-related complications—such as difficult intubation with necessity of awake fiberoptic intubation, infection, need for blood transfusions, respiratory and cardiac problems, and renal dysfunction postoperatively (Puvanesarajah et al., 2017). Therefore, assessment of medical comorbidities, associated with both the disease and the treatment of AS is an important component of the medical clearance before surgery.

Cardiovascular Concerns: According to the American Heart Association and American College of Cardiology (ACC/AHA) cardiac risk assessing guidelines in preoperative surgical preparation of patients with AS, cardiovascular disease is advanced if functional capacity is not reached to a minimum of four Metabolic Equivalents (METS) (Fleisher et al., 2014). These patients with poor functional capacity (<4 METS) are at greater cardiac risk (Goodman and Bass, 2018).

Pulmonary Concerns: Significant spinal deformity in AS can result in restrictive respiratory physiology, with notable decreases in vital lung capacity (Berdal et al., 2012). This may make ventilation during surgery challenging, especially when a prone position is anticipated. For those patients, aggressive pulmonary hygiene in the postoperative period can be encouraged

Neuromuscular Concerns: A thorough physical examination should be performed to document any preoperative sensory or motor deficits, especially in patients undergoing spinal surgery. Notation of any motor weakness is important as this may affect the plan for intraoperative neuromonitoring and the anesthesia (Sciubba et al., 2008).

Renal Concerns: Since AS patients receive non-steroidal anti-inflammatory drugs (NSAID) for a long period, interstitial nephritis may be present due to NSAID use. Advanced AS may be associated chronic kidney dysfunction due to amyloidosis, IgA-nephropathy, and tubulointerstitial nephritis (Ye et al., 2019).

Bone loss Concerns: Taking into consideration that osteoporosis is a common complication of AS, with an incidence between 18.7% and 62%, all patients undergoing spinal surgery should be treated before and after surgery for osteoporosis (van der Weijden et al., 2012).

According to the Assessment of SpondyloArthritis International Society/European League Against Rheumatism (ASAS-EULAR) management suggestions for AS, NSAIDs are considered the first-line drug treatment. To prevent further progression of the disease and achieve remission with improved quality of life, disease-modifying antirheumatic drugs (DMARD) are required: synthetic (such as sulfasalazine) and biological (anti-TNF-α agents such as infliximab, etanercept, adalimumab, golimumab, etc. as well as inhibitors of IL-17-secukinumab) (van der Heijde et al., 2017).

Taking into account the need for chronic therapy and associated side effects of those drugs, patients undergoing surgery need dosage adjustment.

NSAIDs: The perioperative use of NSAIDs or aspirin may be associated with bleeding complications. This is because NSAID-induced inhibition of COX‐1 with reduction of thromboxane A2, which, in turn, will decrease vasoconstriction and platelet aggregation and increase the bleeding time. This effect is reversed only after drug withdrawal. Considering the average half-life of an NSAID, they must be withdrawn at some point prior to surgery. (Franco et al., 2017). On the other hand, in patients, whose cardiovascular risk exceeds the intraoperative benefit (for example, patients with cardiac stents), it’s recommended to continue the aspirin during the perioperative period (Oscarsson et al., 2010; Gerstein et al., 2015). In such situations, the surgeon must be alerted and prepared for bleeding during surgery, even though cardiac doses of aspirin, in general, are not associated with a significant risk of bleeding (Vetter et al., 2014). Since COX-2 inhibitors have little effects on platelet function, there is no need to suspend them during the perioperative period. (Leese et al., 2000; Franco et al., 2017). However, this group of drugs is associated with the risk of significant cardiovascular adverse outcomes.

Glucocorticosteroids (GCS): Patients continuously taking GCS are at risk of intraoperative hemodynamic instability and postoperative infection. A few small randomized controlled trials and systematic reviews demonstrate that there are no significant hemodynamic changes between patients receiving a daily dose of GCS compared to those receiving “stress-dose steroids” preoperatively. (Goodman et al., 2017). Therefore it’s recommended to use the usual daily dose of GCS or prednisone less than 20 mg/day, rather than the “stress dose” with regards to infection risk (Somayaji et al., 2013).

DMARDs: Non-biologic synthetic DMARDs are recommended to continue at the time of surgery since it was shown that the risk of infections is not increased. Perioperative continuation of DMARDS also decreases the risk of exacerbation of the main disease after the surgery (Goodman et al., 2017).

Systematic reviews and meta-analyses of biologic DMARDs revealed an increased risk of serious infections. Most commonly the respiratory and urinary tract infections, skin, and opportunistic infections are developed, including hepatitis B and C, tuberculosis, and various fungal infections such as histoplasmosis (Nard et al., 2015).

Therefore, all current biologic DMARDs should be withdrawn before the surgery and it is important to schedule the procedure after the active period of these drugs (Goodman et al., 2017).

In conclusion, proper perioperative management, consisting of preoperative patient planning, as well as intraoperative and postoperative patient monitoring and care, are required. Further investigations are needed to build up a reliable, evidence-based strategy of effective perioperative management of AS patients undergoing spine surgery.

## Author Contributions

AZ and KG contributed to the writing of the manuscript.

## Conflict of Interest

The authors declare that the research was conducted in the absence of any commercial or financial relationships that could be construed as a potential conflict of interest.
